# Construction of a high-density genetic map and mapping of a spike length locus for rye

**DOI:** 10.1371/journal.pone.0293604

**Published:** 2023-10-30

**Authors:** Yonghe Che, Yunjie Yang, Yanping Yang, Lai Wei, Juan Guo, Xinming Yang, Xiuquan Li, Weihua Liu, Lihui Li

**Affiliations:** 1 Hebei Key Laboratory of Crop Stress Biology, Qinhuangdao, Hebei, China; 2 College of Agronomy and Biotechnology, Hebei Normal University of Science and Technology, Qinhuangdao, Hebei, China; 3 Institute of Crop Sciences, Chinese Academy of Agricultural Sciences, Beijing, China; Jeju National University, REPUBLIC OF KOREA

## Abstract

Genetic maps provide the foundation for QTL mapping of important traits of crops. As a valuable food and forage crop, rye (*Secale cereale* L., RR) is also one of the tertiary gene sources of wheat, especially wild rye, *Secale cereale* subsp. *segetale*, possessing remarkable stress tolerance, tillering capacity and numerous valuable traits. In this study, based on the technique of specific-locus amplified fragment sequencing (SLAF-seq), a high-density single nucleotide polymorphism (SNP) linkage map of the cross-pollinated (CP) hybrid population crossed by *S*. *cereale* L (female parent) and *S*. *cereale* subsp. *segetale* (male parent) was successfully constructed. Following preprocessing, the number of 1035.11 M reads were collected and 2425800 SNP were obtained, of which 409134 SNP were polymorphic. According to the screening process, 9811 SNP markers suitable for constructing linkage groups (LGs) were selected. Subsequently, all of the markers with MLOD values lower than 3 were filtered out. Finally, an integrated map was constructed with 4443 markers, including 1931 female mapping markers and 3006 male mapping markers. A major quantitative trait locus (QTL) linked with spike length (SL) was discovered at 73.882 cM on LG4, which explained 25.29% of phenotypic variation. Meanwhile two candidate genes for SL, *ScWN4R01G329300* and *ScWN4R01G329600*, were detected. This research presents the first high-quality genetic map of rye, providing a substantial number of SNP marker loci that can be applied to marker-assisted breeding. Additionally, the finding could help to use SLAF marker mapping to identify certain QTL contributing to important agronomic traits. The QTL and the candidate genes identified through the high-density genetic map above may provide diverse potential gene resources for the genetic improvement of rye.

## Introduction

Rye (*Secale cereale* L., 2*n* = 2*x* = 14), a cross-pollinated relative of common wheat with an R genome, carries valuable genes associated with high protein content, disease resistance, drought/cold tolerance, and yield improvement [1]. It is grown in central and eastern Europe, which are the main rye cultivation regions in the world [2]. Furthermore, rye is commonly found in colder northern regions of China. This crop species is known to possess a wide range of disease resistance genes that confer resistance against wheat susceptibility diseases, including stripe rust and powdery mildew. These disease resistance genes present in rye can serve as valuable genetic resources for the improvement of wheat, thereby promoting sustainable agricultural practices. Over the years of research, a series of progress has been made in introducing rye disease resistance and superior agronomic traits into wheat crops by using distant hybridization and chromosome engineering technology [3–6]. In summary, rye represents a vital gene resource donor for enhancing disease resistance, stress tolerance, and agronomic characteristics in wheat breeding programs, while also serving as an exceptional food and fodder crop.

Plant wild germplasm resources frequently exhibit significant genetic diversity; however, due to environmental changes and human interventions, wild species have faced ongoing reductions in their populations. The more noteworthy it is that a wild species called *Secale cereale* subsp. *segetale*, was found at Xinjiang Province of China, especially considering that rye does not originate from this region. This unique rye from China is classified as a subspecies of *S*. *cereale* [7]. *S*. *cereale* subsp. *segetale* shows rich genetic diversity in traits such as plant height, spike morphology, grain size, stress tolerance, disease resistance and tillering ability [8]. In addition, compared to common wheat, *S*. *cereale* subsp. *segetale* has a higher lysine content [9]. Gliadin study has revealed that *S*. *cereale* subsp. *segetale* showed high genetic diversity both among and within populations, which makes it suitable for population diagnostics [10]. Some researchers have reported that cultivated rye was directly domesticated from weedy rye *S*. *cereale* subsp. *segetale* [11].

In recent years, the utilization of next-generation sequencing (NGS) technology for the identification of single nucleotide polymorphism (SNP) markers has emerged as an appropriate and effective approach for the construction of high-density genetic maps in plants. The application of mapping the double pseudo-crossing (cross-pollinated, CP) hybrid population has contributed to discover effective QTL in self-incompatibility population [12–14]. The SLAF-seq method, which employs a twofold pseudo-test cross procedure, is a high throughput genome sequencing technology [15]. To date, SLAF-seq has been used effectively to create high-density genetic maps and investigate the genomes among various species using SNP markers, such as Caixin and Zicaitai (*Brassica rapa*) [16], *Elymus sibiricus* [17], *Vitis vinifera* L. [18], and *Agropyron* Gaertn. [19]. Therefore, the application of SLAF-seq technology to develop SNP markers may facilitate the construction of a high-quality, densely populated genetic linkage map for rye, which will be a valuable tool for QTL mapping, particularly for complex quantitative traits such as tiller number, plant height, and spike length.

The initial QTL mapping studies in rye were based on the agronomic trait performance of lines and RFLP maps of F_2_-derived mapping populations [20, 21]. Marker-assisted creation of introgression line libraries was applied to approach QTL regulating agronomic characteristics traits in rye genetic resources [22, 23]. Subsequent QTL mapping research focused on the dominant dwarfing gene *Ddw1* [24], in vitro response [25], α-amylase activity and associated traits [26–28], and morphological rye traits [29]. The first complete investigation identifying QTL of yield and quality-related traits was undertaken on progenies of two elite bi-parental mapping populations within the ’Petkus’ gene pool [30]. The genetic diversity in elite rye germplasm, as well as the F_2:3_ testcross design, facilitates rapid QTL mapping to identify genes regulating grain production, grain weight, and tillering [31]. The first genome-wide association study (GWAS) was used for analyzing plant height, germination, grain quality and yield of rye hybrids [32]. Spike length (SL) is not only a crucial aspect of the plant type of grasses, but also a key spike trait that is strongly correlated with grain yield. Therefore, identifying and mining the genetic loci of quantitative traits related to SL will significantly enhance the molecular breeding efficiency of rye and wheat crops. At present, limited reports focus on the SL QTL in rye.

In this study, we employed the SLAF-seq method for rapid discovery of SNP markers for rye. These newly developed markers were utilized to construct a high-density genetic map of a CP hybrid population crossed by *S*. *cereale* and *S*. *cereale* subsp. *segetale*, and the preliminary QTL for spike length were mapped. The screening of SNP markers with potential functions in our study could provide a new genomics and breeding basis for rye and related cereal crops.

## Materials and methods

### Plant materials and phenotyping of traits

The rye parents were collected from the plant resources investigation group of the Chinese Academy of Agricultural Sciences (CAAS), Beijing, China. Seeds and specimens of parents are currently maintained in the National Center for Crop Germplasm Conservation of CAAS. The agronomic significance and contrasting characteristics of these two parent materials, such as plant height, spike length, and number of spikelets, led to their selection. During May–June 2018, the CP hybrid population was generated through manual pollination between *S*. *cereale* Z1672 (female parent) and *S*. *cereale* subsp. *segetale* 89R41 (male parent) at the farm of Hebei Normal University of Science and Technology in Qinhuangdao (119°15′E, 39°72′N), Hebei, China. In October 2018, the CP hybrid population seeds were germinated in the greenhouse and the seedlings were transplanted to the experimental field subsequently. Young healthy leaves were collected from the two parents and the 158 population individuals, stored in liquid nitrogen and then transferred to freezer at -70°C before sent to company for further analysis.

### Library construction, Illumina sequencing, and data filtering

An improved SLAF-seq strategy [33] was used for library construction in this research. Initially, the SLAF-seq quality reference, *Oryza sativa* L. *japonica*, was utilized to design maker discovery tests by simulating in *silico* the quantity of markers generated by various enzymes. Following, the genomic DNA of the two parents and the CP population was digested using the enzyme *Rsal* (New England Biolabs, NEB, USA). Subsequently, sequencing adapters labeled with a single nucleotide (A) and duplex tag-labeled (PAGE-purified; Life Technologies, USA) were added to the digested fragments, followed by PCR amplification. Finally, the product above was excised and purified (Qiagen, Hilden, Germany) to obtain fragments ranging from 464 to 484 bp. In addition, paired-end sequencing (125 bp per end) was performed on an Illumina HiSeq 2500 system (Illumina, Inc; San Diego, CA, USA). The same procedure was used to extract genomic DNA from *Oryza sativa* L. *japonica* as a control sample to evaluate the accuracy of library construction and sequencing experiments.

### Sequence data grouping and genotyping

The methods outlined by Sun [33] were used to genotype and organize the SLAF-seq data. Low-quality reads (quality score <20e) were removed, and raw readings were allocated to 158 individual samples based on the duplex barcode sequences. High-quality readings with quality scores (QC) greater than 30 were retained for next analysis. The SOAP program [34] was used to align these high-quality reads onto the Weining rye genomic [35] sequence, and the GATK software kit was used to SNPs detection between two parents and offspring. The detailed process can be found on GATK’s official website, https://www.broadinstitute.org/gatk/guide/best-practices?bpm=DNAseq#variant-discovery-ovw. One SLAF locus was defined as a set of sequences that map to the same location [36]. SNP loci between the two parents were detected, and SLAF makers above three SNPs were discarded. Each of the SLAF loci’s alleles was identified in both the parental and offspring SNP loci, ensuring all polymorphism SLAF loci were genotyped consistently. Then, based on parental reads with sequence depths more than ten-fold, the alleles of each SLAF locus were determined, and each genotype sequence was required to contain at least 30% of offspring information. Rye, as a kind of diploid cross-pollinated species, has seven segregation types in its maker code for polymorphic SLAFs. Markers from the segregation pattern of aa × bb were filtered out because the map was constructed using the CP population derived from two heterozygous parents.

Genotyping scoring was conducted using a Bayesian technique as outlined by Sun [33] to ensure the quality of the genotyping. The following criteria were used to select high-quality SLAF markers for the genetic mapping project. Markers with more than 30% missing data and SNPs with average sequences depths less than two-fold in every offspring and four-fold in parents were eliminated. Then markers exhibiting significant segregation distortion (*P* <0.01) based on the chi-square test were originally omitted from the genetic map development but included as accessory markers.

### Genetic linkage map construction and mapping of SL locus

Based on their positions on the Weining rye genome [35], marker loci were largely divided into linkage groups (LGs). To confirm the stability of each LG’s markers, modified logarithm of odds (MLOD) scores was computed and markers having MLOD scores less than three were deleted prior to ordering. The HIGH MAP approach was used to organize the SLAF markers in a precise order and rectify genotyping mistakes within LGs in order to guarantee efficient development of a high-quality map [37]. Following that, the SMOOTH error correcting technique was utilized based on parental genotype contribution [38], followed by the imputation of missing genotypes by the K-nearest neighbor algorithm [39]. Skewed markers were then added to this map using a multipoint maximum probability approach. The Kosambi mapping function was used to calculate map distances [40].

The quality of genetic maps was evaluated using various methods including individual completeness, haplotype maps, heat maps, and spearman’s rank correlation coefficient. The recombination relationship between markers of each LG was evaluated using a heatmap. Collinearity blocks within each LG were detected by comparing with the Weining rye genome [35].

QTL analysis of SL was identified by R/qtl software [41, 42]. Using 1000 permutation tests at a significance level of *P* <0.05 and a mapping step of 1.0 cM, the LOD threshold for a QTL to be declared significant was computed. Then, this LOD threshold value was utilized to find QTL that were strongly related with the trait. The contribution rate of QTL in each 1 cM interval in each LG was calculated.

By comparing with the genome of Weining rye, multi-database gene function annotation was performed on genes within the localization interval. Expression data for different tissue periods of rye can be downloaded from the WheatOmics database [43] (http://wheatomics.sdau.edu.cn/) on the wheat multi-omics website, and the TPM (Transcript per million) was used as the unit of expression level, and the gene expression heatmaps were generated using TBtools software [44].

## Results

### Analysis of SLAF-seq data and SLAF markers

**Fig 1:**
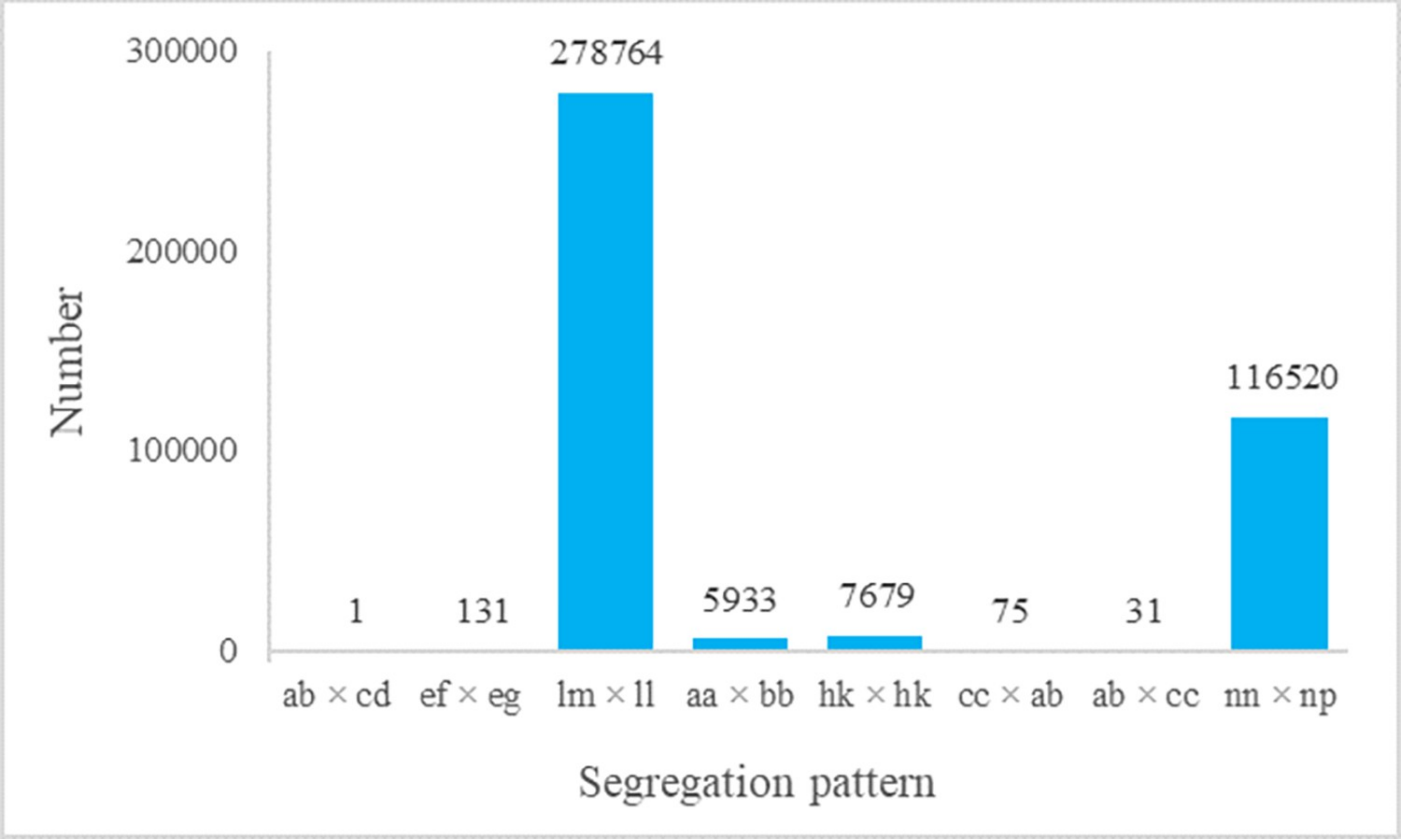
Numbers of markers for eight segregation types. The x-axis represents eight segregation patterns of polymorphic SNPs markers; while the y-axis indicates the number of markers.

After preprocessing, a total of 1035.11 M reads were generated by high-throughput sequencing. On an average, Q30 (quality scores of at least 30) was 89.75% and the GC content was 46.59%, which together resulted in high-quality source data. To confirm the accuracy of the SLAF library creation, the control sequencing data was assessed. *Rsal* enzyme was used for the SLAF library construction. For the control in this study, the ratio of paired-end mapped reads was 84.99% and the percentage of digestion normally was 90.43%. The construction of the SLAF library was successful, as evidenced by a total of 2425800 SNPs were detected in these reads, of which 409134 could be successfully genotyped and 403201 could be used for genetic map construction (Fig 1). Low-quality SNPs were filtered out with a sequencing depth of less than 4 and a bias segregation of less than 0.01 in the parent. Ultimately, 9811 SNP makers were used for construction of rye genetic map, including 3444 markers from the segregation pattern of np × nn, 5352 markers from lm × ll, 1010 markers from hk × hk, and 5 markers from ef × eg.

### High-density genetic map construction and its basic characteristics

Out of the 9811 markers, based on MLOD scores ≥3 between the two high-quality SNP markers, 4443 SLAF markers were finally obtained for genetic map construction. By comparing with the reference genome (Weining V1: https://ngdc.cncb.ac.cn/gwh/Assembly/12832/show), the SLAF markers were divided into seven linkage groups representing the seven rye chromosomes. Eventually, among the markers, 1931were mapped on the female (*S*. *cereale* Z1672), 3006 on the male map (*S*. *cereale* subsp. *segetale* 89R41), and 4443 on the integrated map. All the information of markers on the integrated map was provided in S1 Table, including the marker name, LGs, genetic position, and physical location on the rye genome. The lengths of the female, male, and integrated genetic maps were 892.37, 1282.15, and 1112.54 cM, respectively. In the female, male, and integrated maps, the average distance between makers was 0.47, 0.43, and 0.25 cM, respectively. The average depth of the markers was 111.49 × in the parents, and 16.79 × in the offspring.

**Fig 2:**
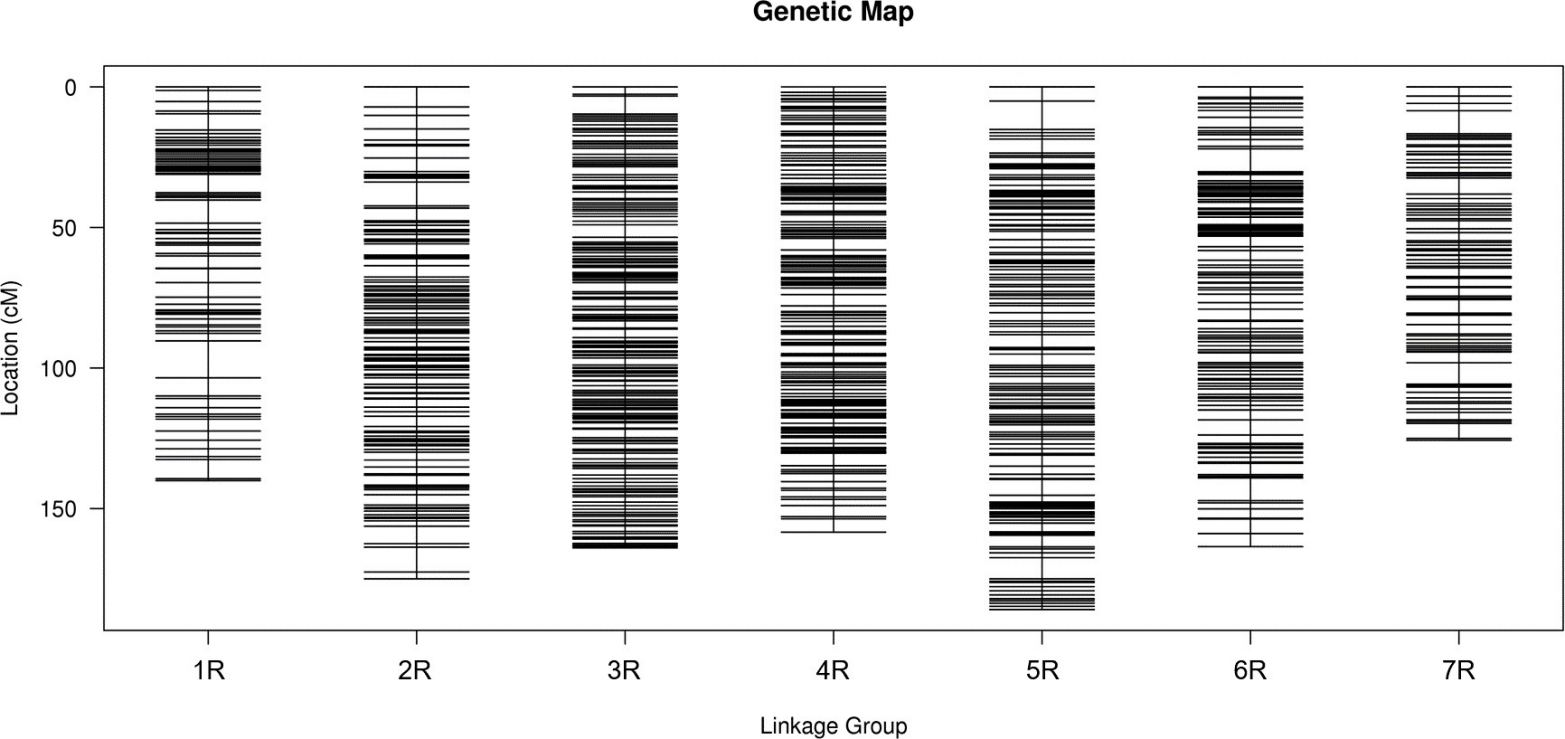
The integrated high-density genetic map of rye based on 4443 SNPs. The x-axis and y-axis represent linkage groups and location, respectively.

For the integrated map, the number of SNP markers on each LG ranged from 497 to 809 (Table 1 and Fig 2). LG5 showed the largest LG allocated 809 makers and a genetic map distance of 185.91 cM, with an average gap between markers of 0.23 cM. LG7 was the shortest LG included 500 markers and a total length of 125.66 cM, with an average distance of 0.25 cM. Moreover, LG4 was the most saturated, with 745 markers of an average distance of 0.21 cM. LG1 and LG2 were the sparsest with an average density of 0.28 cM, consisting of 497 and 633 markers, respectively. LG3 had 638 markers with a genetic map distance of 163.99 cM and an average distance 0.26 cM. LG6 had 621 markers with a genetic distance of 163.54 cM and an average distance 0.26 cM.

**Table 1:** Summary statistics of the rye map.

Chromosome ID	Marker Num	Total Distance (cM)	Average Distance	Gaps <5 cM (%)	Max Gap	Number of markers of segregation distortion	Segregation distortion ratio (%)
1R	497	140.06	0.28	98.59	13.12	30	6.04
2R	633	174.99	0.28	99.37	8.84	17	2.69
3R	638	163.99	0.26	99.84	6.43	1	0.16
4R	745	158.39	0.21	100.00	4.74	1	0.13
5R	809	185.91	0.23	99.50	10.10	89	11.00
6R	621	163.54	0.26	99.35	8.16	11	1.77
7R	500	125.66	0.25	99.20	8.24	115	23.00
Total	4443	1112.54	0.25	99.41	13.12	264	5.94

A total of 4443 markers were used to construct the genetic map, of which 264 were segregation distortion markers, accounting for 5.94% of the total. Among the LGs, LG7 are exhibited the largest number of segregation distortion markers, with 115 markers (Table 1).

### Evaluation of the genetic map

**Fig 3:**
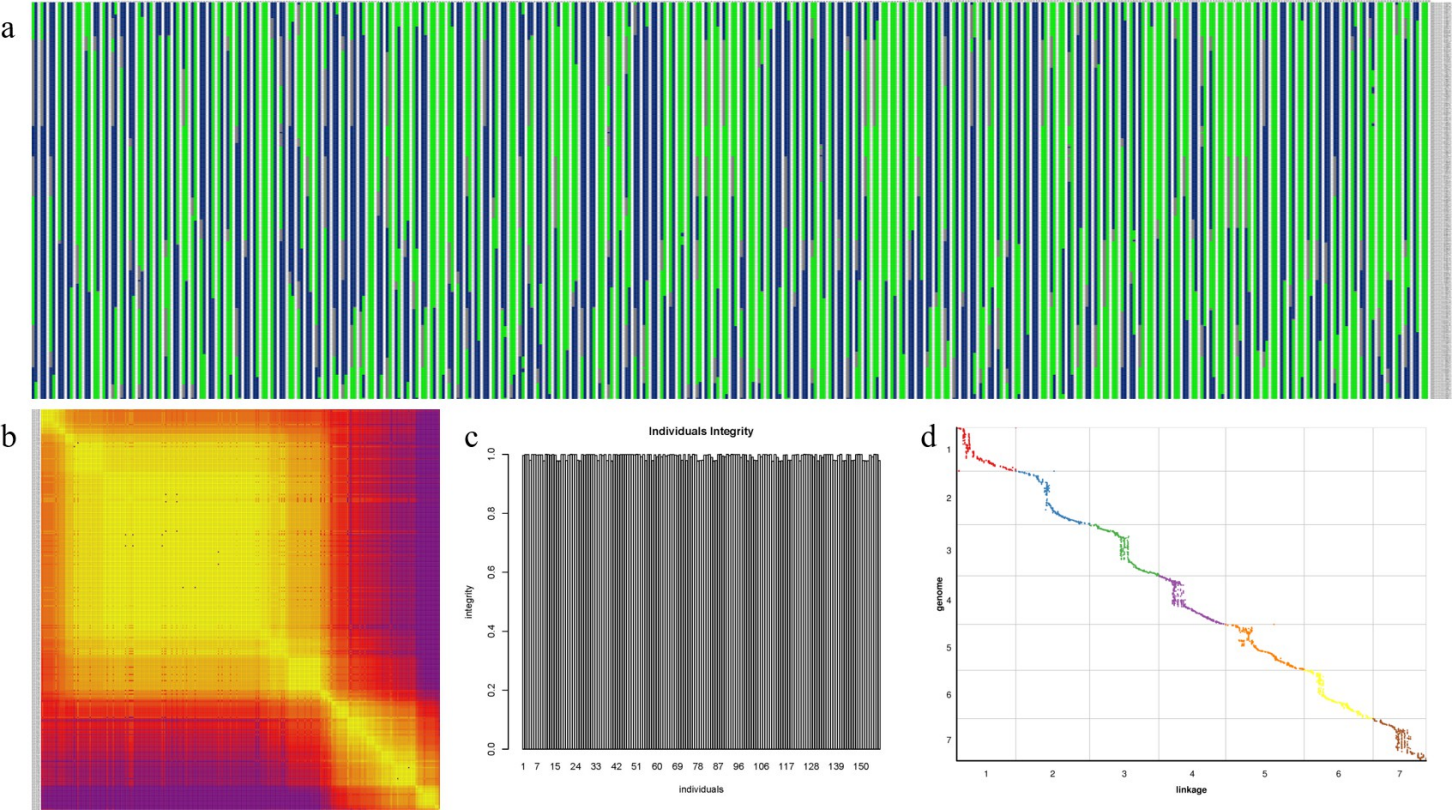
Evaluation of the genetic map. (a) haplotype maps for each individual of LG4; (b) heatmap of integrated group LG4; (c) average integrity of mapping markers; (d) collinearity analysis between the linkage map and the corresponding physical map.

Using 4443 SLAF markers, haplotype map was produced for each individual, and revealing many of the reassembly blocks (Fig 3A). In addition, Heatmap results showed significant correlations in linkage groups between nearby markers (Fig 3B). The mapped markers’ average integrity was 99.33%, indicating the genetic map’s comparatively high quality (Fig 3C). The linkage map and related physical map were examined for collinearity analysis using the Weining rye genome as a reference, and the spearman coefficient was approximately 1 (Fig 3D). These results indicate the high quality of the present genetic maps.

### Quantitative trait loci associated with SL

**Fig 4:**
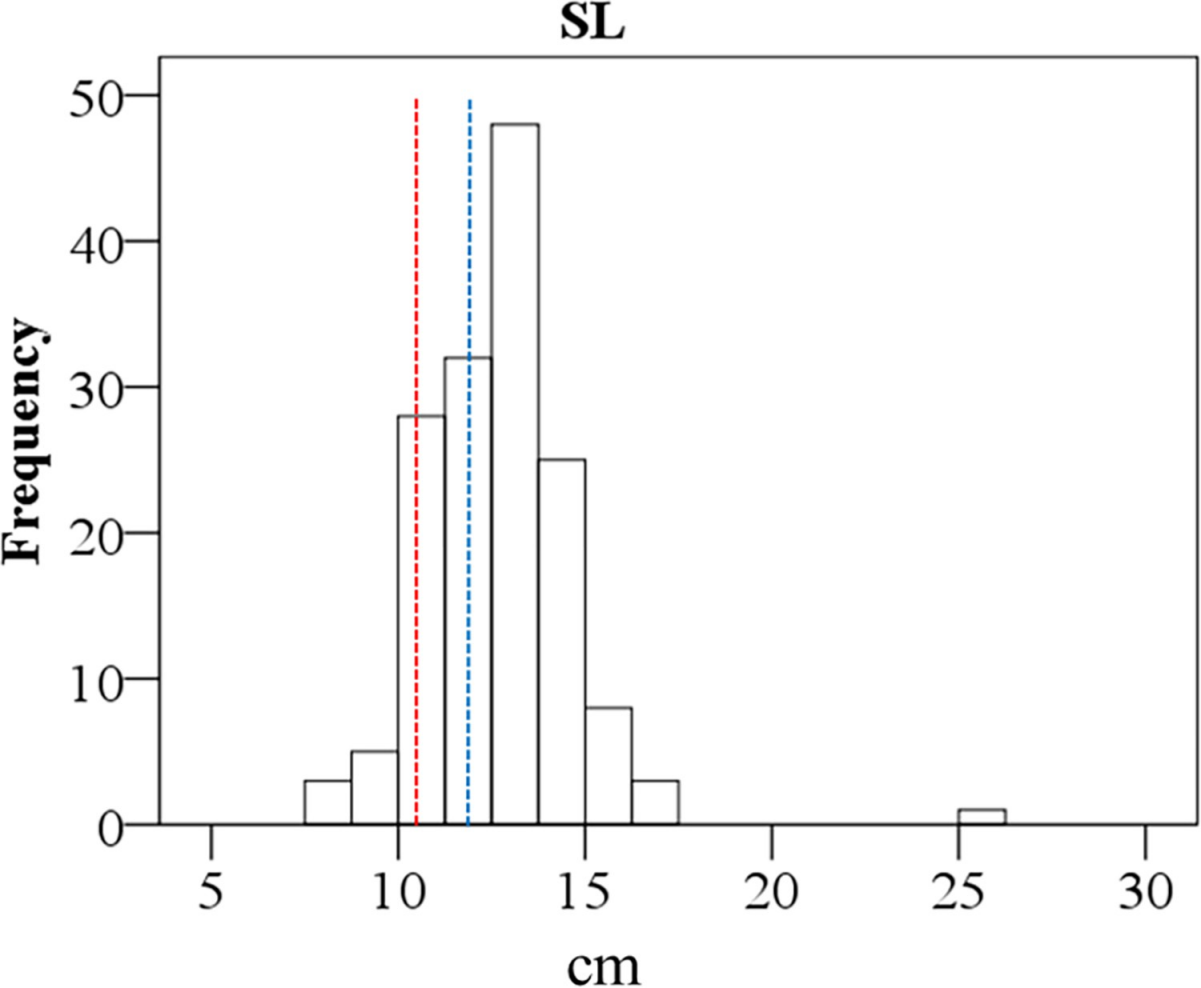
Frequency distribution of the traits SL in CP population. Dashed lines mark the means of parents (red = Z1672, blue = 89R41).

Phenotypic data were generated for SL from the complete set of CP population. The ranges of SL were 7.50–25.31 cm, with a mean value of 12.66±1.93 cm (Fig 4).

**Fig 5:**
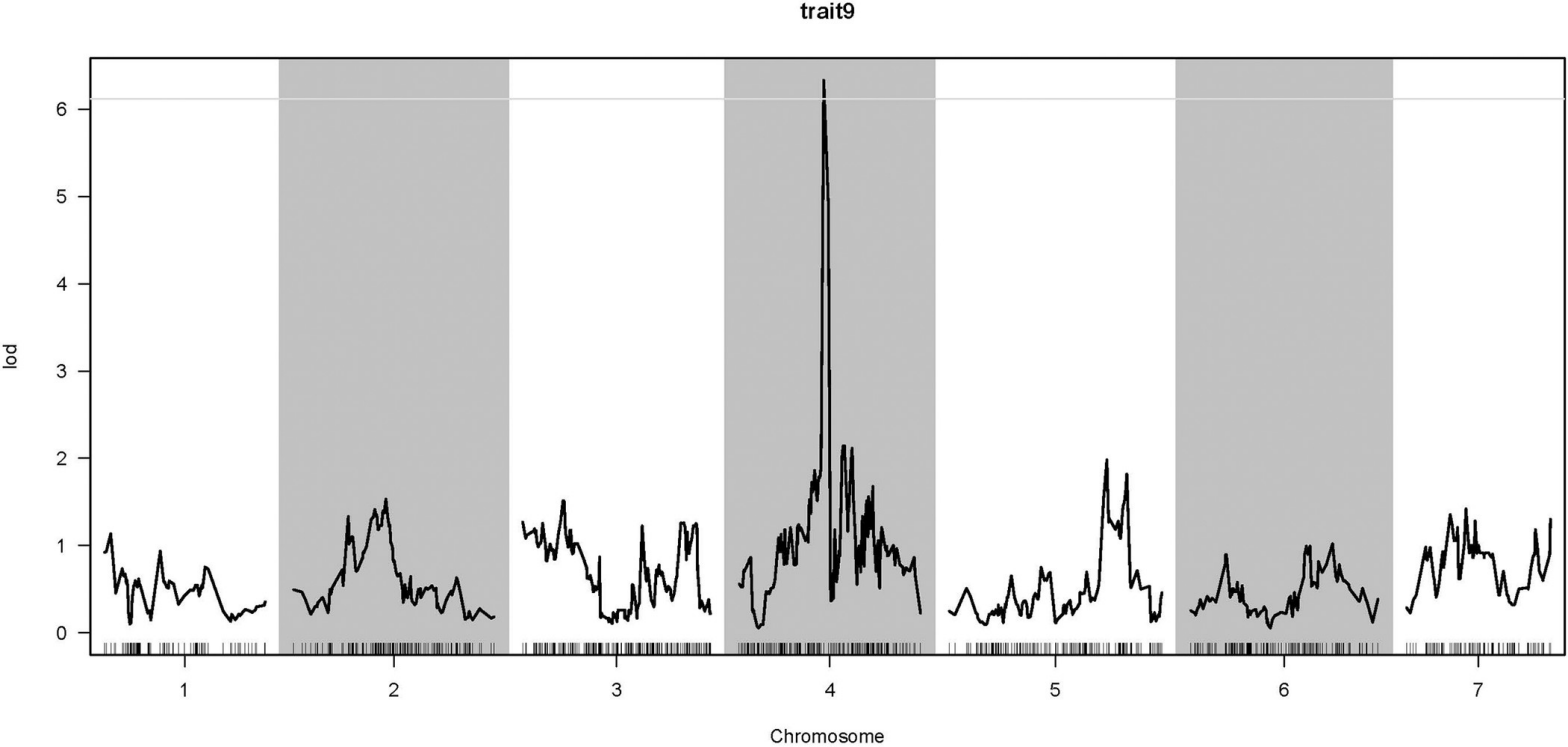
QTL analysis of SL on linkage groups. The x-axis indicates the linkage groups and the marker order. The horizontal gray line indicates the threshold of the LOD score (6.11) for significance (*P* = 0.1) at the whole genomic level.

**Fig 6:**
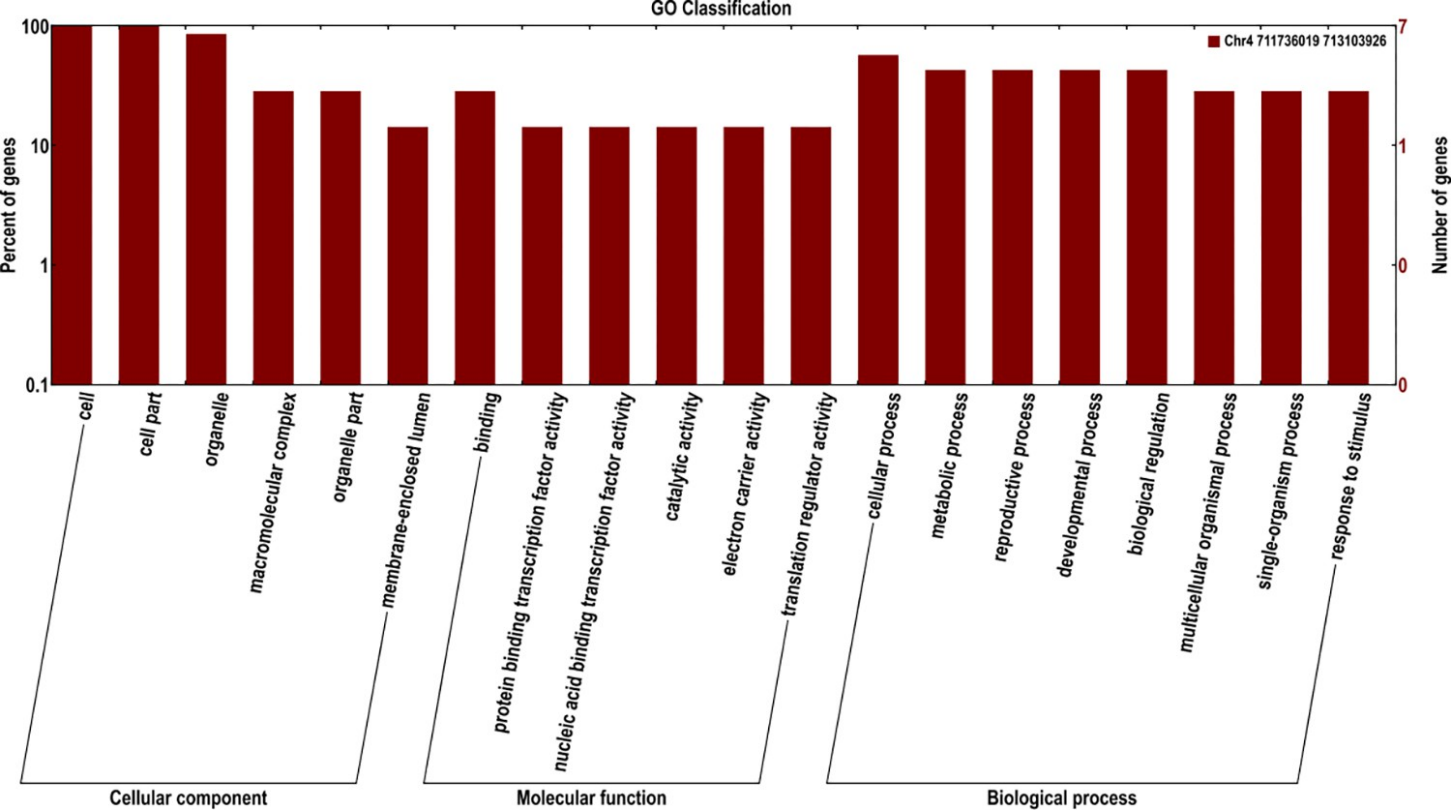
GO annotated results of genes in candidate regions.

Using R/qtl software for the QTL linkage analysis of SL, the LOD threshold was determined to be 6.11 (Fig 5). A major QTL for SL was mapped at 73.882 cM on LG4, explaining 25.29% of the phenotypic variation. Three SNP markers (Marker 145702, Marker145685 and Marker145635) were uncovered within the flanking region of the SL QTL. In the mapped QTL segment, nine candidate genes (*ScWN4R01G328800*, *ScWN4R01G328900*, *ScWN4R01G329000*, *ScWN4R01G329100*, *ScWN4R01G329200*, *ScWN4R01G329300*, *ScWN4R01G329400*, *ScWN4R01G329500*, and *ScWN4R01G329600*) were detected. By the COG, GO, KEGG, Swissport and Nr databases, there were five, seven, four, nine and nine genes, respectively annotated (S2 Table). Seven genes (*ScWN4R01G328900*, *ScWN4R01G328800*, *ScWN4R01G329300*, *ScWN4R01G329000*, *ScWN4R01G329500*, *ScWN4R01G329600*, and *ScWN4R01G329200*) annotated by GO database were involved in biological process and cellular component (Fig 6).

**Fig 7:**
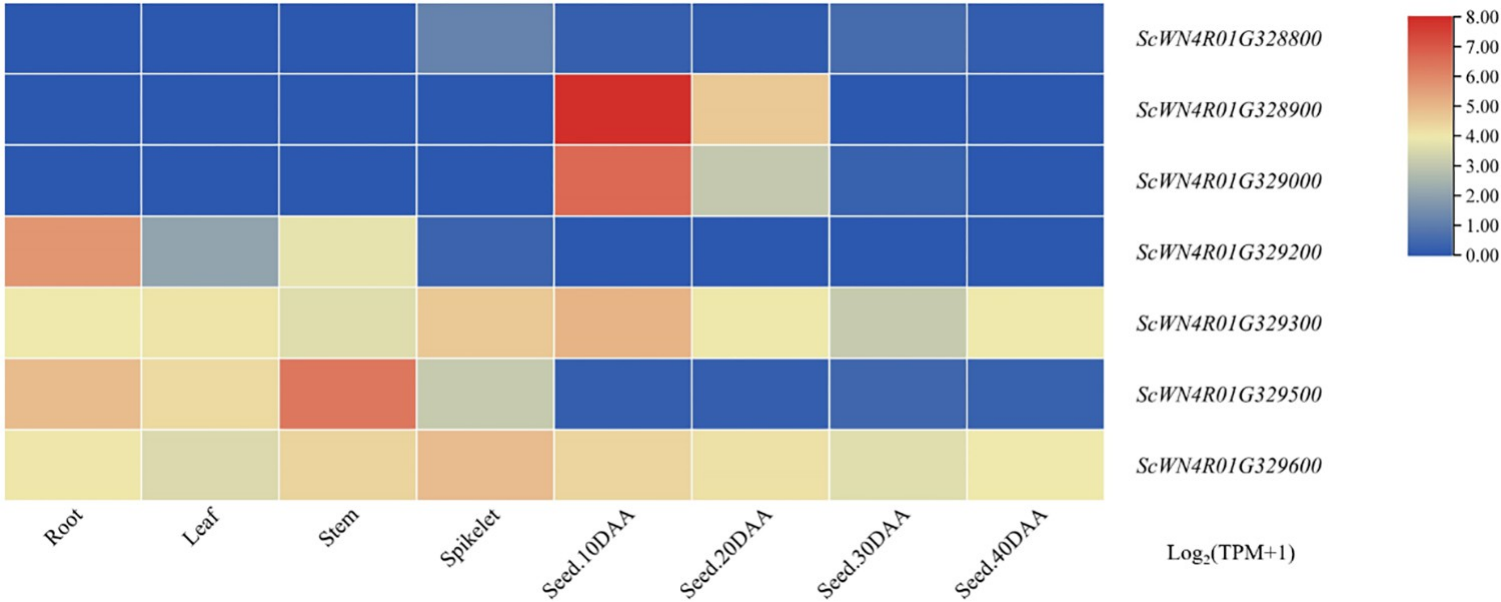
Expression patterns of candidate genes in the root, stem, leaf, spike and developing grain tissues of Weining rye. Root, stem, leaf and spike samples were collected at the heading stage. The grain samples were collected at 10, 20, 30 and 40 d after anthesis (DAA), respectively. The data of expression profiles were obtained from the public online database (http://wheatomics.sdau.edu.cn/expression/rye.html).

In public databases, by searching the RNA-seq data of these nine candidate genes under non-stress growth conditions, their expression in root, stem, leaf, spike and grain was analyzed (Fig 7). Two of the genes, *ScWN4R01G329300* and *ScWN4R01G329600*, were expressed significantly higher in spike than other genes, and showed specificity among tissues. These two genes could be candidate genes for SL and need to be further verified in future work.

## Discussion

### Construction of a high- density genetic map for rye based on SLAF-seq

Rye serves as a valuable gene donor for wheat breeding due to its superior agronomic characteristics, disease resistance, and environmental adaptability [45–48]. Some excellent wheat varieties have been created as a result of the successful introduction of rye’s superior traits into wheat through distant hybridization and chromosome engineering, including the 1RS/1BL translocation line with stripe rust resistance [49, 50], and the 6R addition line WR49 and 1R addition line N9436B that carry rye powdery mildew resistance [51, 52]. The release of the Weining and Lo7 rye genome data [35, 53] has greatly advanced the gene mining research on related rye. For instance, a novel leaf rust resistance gene was identified in rye with the reference genome [54], the candidate regions and genes for nine agronomic and quality traits of rye were predicted [32]. As the advent of the era of rye genomics, there will be more research reports on rye genome.

Genetic maps play an important role in QTL mapping studies of agronomic traits and molecular improvement breeding. Constructing high-quality, high-density genetic maps requires lots of reliable molecular markers. In previous research on rye, researchers mainly carried out genetic diversity analysis, genetic variation analysis, genetic linkage mapping and excellent gene mapping and discovery by developing markers such as RAPD [55], AFLP [56] and SSR [57]. However, these markers were limited in their number, which restricted future development of high-density physical maps.

With the development of high-throughput genome sequencing technology, SLAF-seq technology has emerged as a powerful tool for developing numerous SNP markers in various species genomes, thereby playing an increasingly important role in plant genetics and breeding research. For example, a set of competitive allele-specific PCR (KASP) markers targeting 14 individual rye chromosomal arms has been built using SLAF-seq data from five rye cultivars [58], and a physical map of the stripe rust resistance gene on rye chromosome 6R has been constructed using SLAF-seq technology [59]. In this study, we constructed a SNP marker-based genetic map of rye using 158 individual plants of the rye CP hybrid population, with an average of 634 markers per LG and an average distance of 0.25 cM. Compared to current references, this present rye map represents the densest genetic linkage map of rye. However, this map is not saturated yet and further work will be needed in the future to fill several significant gaps in the 7 LGs with distances between 9.75 and 35.30 cM. Nonetheless, the present rye genetic map lays a foundation for tapping elite genes in wild *segetale* rye and supply a reference for related plant study.

### Quantitative trait loci analysis of SL

SL is a crucial agronomic trait that influences the yield per unit area of wheat crops. Therefore, investigating and identifying QTL associated with SL is of great significance for genetic research and molecular-assisted breeding of rye and wheat crop yield components. In this study, SLAF markers were associated with the SL trait, and a major QTL involved in SL was detected at 73.882 cM on LG4, which preliminarily confirmed that LG4 was a potential SL related chromosome in rye. Previously, a study has reported the existence of QTL related to SL on chromosomes 2, 4, 5 and 6 in rye [29]. However, our study is different from the interval mapped on chromosome 4 in previous study, which may be a new QTL regulating SL. Existing studies have shown that the genetic characteristics of grain development traits are the presence of QTL loci on one or more chromosomes, and the number of QTL on each chromosome is also different. For example, in millet seven QTL related to SL identified on chromosomes 1, 2, 4, 5 and 9 [60], eight QTL related to SL on chromosomes 2, 5, 6, 7 and 8 [61]; two stable QTL for SL were identified on chromosomes 2H and 7H in barley [62]; in wheat, nine SL QTL detected using a 9K SNP chip, and explained the highest contribution rate of 23.60% [63], fifty-one significant SNP loci associated with SL were detected by genome-wide association analysis [64], and 30 SL QTL revealed through a genetic linkage map constructed from SSR and SNP markers [65].

The abundant genetic variation for important agronomic traits in the rye CP hybrid mapping population were observed, which allowed researchers to use SLAF marker mapping to identify QTL loci that contributed to important agronomic traits. Once SLAFs strongly correlated with the target trait were obtained, primers could be designed for PCR testing of the target trait. Additionally, aligning SLAF sequences associated with traits with the genome sequences facilitates the identification of rye gene resources for wheat improvement and rye gene cloning.

In this study, the high-density genetic map constructed supposed to provide a robust tool for further basic and applied research in rye. However, due to the self-incompatibility of cross-pollination in rye, it is challenging to construct a stable mapping population and conduct multi-year and multi-site QTL mapping. How to improve or solve this issue is a future research focus.

## Conclusions

In conclusion, we constructed a high-density genetic map based on simplified genome sequencing data of 158 individuals in a CP hybrid population. The map consisted of 4443 high-quality SNP markers spanning 1112.54 cM on seven chromosomes. Based on this map, one QTL relating to spike length was initially identified. Through the analysis of sequence and expression of candidate genes within the QTL mapping regions, two putative genes were revealed, *ScWN4R01G329300* and *ScWN4R01G329600*, potentially responsible for the target traits.

## Supporting information

S1 Table All information of the constructed genetic map of rye. (XLSX)

S2 Table Multi-data library betting interpretation of the candidate genes. (XLSX)
